# Planning a programme to prevent visual loss from diabetic retinopathy

**Published:** 2015

**Authors:** Allen Foster

**Affiliations:** Professor of International Eye Health, London School of Hygiene & Tropical Medicine

Diabetes mellitus is increasing, so it is relevant to plan services to prevent vision loss from diabetic retinopathy. There are 3 target groups to consider:

- the community
- people known to have diabetes mellitus (DM)
- people with sight-threatening diabetic retinopathy (STDR)

Table 1 shows what eye health workers can do for each group.

**Table 1.** 

Target Group	What eye health workers can do
The community	Health promotion about healthy life style and good nutrition
	Visual acuity testing age 40 onwards
People known to have diabetes	An annual eye examination, including visual acuity testing and examination of the retina (fundoscopy or retinal photographs)
People with sight-threatening diabetic retinopathy (STDR)	Treatment for sight-threatening diabetic retinopathy (STDR)

In order to provide the relevant services that each group needs, it is useful to undertake a situational analysis to set priorities and develop an action plan.

## 1. What is the need?

We must work out how many people need the two key services required to address DR: case detection (screening, i.e. an annual eye examination) and treatment of sight threatening retinopathy (e.g. laser, or medical injections).

- **Case detection.** How many people with diabetes will need an annual eye examination?
- **Treatment.** How many people with sight threatening diabetic retinopathy will need treatment?

In order to answer question 1, we need to estimate:

- The size of the population (over 20 years old) in the country or district (usually available from census records)
- The **proportion** of the population (over 20 years old) with diabetes mellitus (available at **www.diabetesatlas.org**)

Then it is straightforward to calculate the total **number of people** with diabetes who will need an annual eye examination.

In order to answer question 2, we need to estimate:

- The number of people over 20 years old with diabetes (the answer to question 1)
- The **proportion** of people with diabetes who have sight-threatening DR (STDR). A reasonable estimate in low-income countries is 10% of all people with diabetes^1^.

Summarise your answers to questions 1 and 2 in Table 2.

**Table 2.** Key indicators of need

**Case detection**	How many will need annual eye examination?	
**Treatment**	How many will need treatment for sight-threatening diabetic retinopathy?	

## 2. What is being done today?

Knowing what the need for services is, estimate what is being done at present and summarise this in Table 3. Compare with Table 2 to see how much more needs to be achieved.

**Table 3.** Key indicators of service provision

**Case detection**	How many people with diabetes have an eye examination per year?	
**Treatment**	How many people are treated for STDR per year?	

## 3. At what stage is our service provision?

The following activities all help to ensure that everyone with diabetes and STDR are able to access screening and treatment services. They are ordered, starting with activities conducted by the eye health team and then radiating out into the general hospital and eventually into the community.

Which of the following are currently being done, at local and national level?

- Diagnosing and treating patients with STDR attending an eye clinic (with/without outreach clinics)
- Examining all patients with diabetes for STDR at hospital diabetes clinics (with/without outreach clinics)
- Creating a register of all known people with diabetes mellitus and calling people for an annual eye examination
- Identifying people with undiagnosed diabetes and STDR in the community
- Educating health professionals about diabetes and STDR
- Educating the community

## 4. What are the available resources?

- Who are the health professionals able to offer screening services?
- Who are the health professionals able to offer treatment for STDR?
- What equipment is available for diagnosis?
- What equipment is available for treatment?
- What management protocols are available?
- Is there a database of patient records?
- What is the financial support?
- What is the leadership for the service?
- What statistics and information are collected and monitored (e.g. via the hospital's management information system?)
- What patient information materials are available?
- Who are the stakeholders? (Government, non-governmental organisations, the community)

Having completed this situational analysis, you can decide what are the next priorities and what are the important actions that need to be taken to improve the DR service.
